# Catheter Ablation of Pediatric Atrioventricular Nodal Re-entrant Tachycardia

**DOI:** 10.19102/icrm.2020.1100902

**Published:** 2020-09-15

**Authors:** Shunmuga Sundaram Ponnusamy, Giridhar Muthu, Vijesh Anand

**Affiliations:** ^1^Department of Cardiology, Velammal Medical College Hospital and Research Institute, Madurai, Tamil Nadu, India

**Keywords:** AVNRT, pediatric arrhythmia, radiofrequency ablation

## Abstract

Catheter ablation is considered as the standard treatment for all patients with symptomatic drug-refractory tachyarrhythmia. The safety and efficacy of the procedure in the adult population is well-established. Due to the small size of the patient and difficulty in attaining venous access, infants are rarely subjected to radiofrequency ablation. Here, we report a case of drug-refractory AV nodal re-entrant tachycardia in a two-year-old child. Radiofrequency ablation was performed with a 5-Fr sized medium-curve ablation catheter deployed at the slow pathway region where a fractionated A-wave with slow-pathway potential was recorded. After ablation, no recurrence of SVT at the end of 12 months of follow-up was observed.

## Introduction

Although catheter ablation for cardiac arrhythmia has been performed in adult populations since the early 1980s, ablation for cardiac arrythmias in the pediatric population was not attempted until the early 1990s.^1,2^ Supraventricular tachycardias (SVTs) are the most commonly encountered substrates, among which, those related to the accessory pathway account for 75% of cases in older children and 95% of cases among infants. Atrioventricular (AV) node reentrant tachycardia (AVNRT) is a less frequent (12%) occurrence.^3,4^ Most of these arrhythmias respond to medical management and catheter ablation is reserved for drug-refractory cases only. Cryoablation is the preferred modality for infants and young children as it reduces the risk of AV blockage. Herewith, we report the case of a two-year-old child with drug-refractory AVNRT with repeated hospitalization for the arrhythmia who was subsequently treated by radiofrequency (RF) ablation.

## Case report

**Figure 1:**
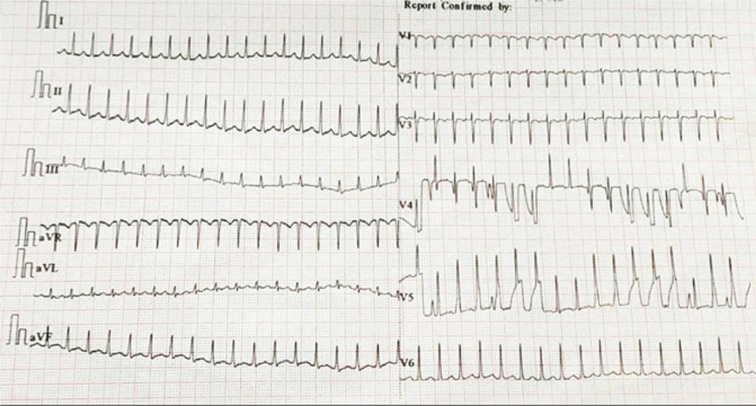
ECG taken during tachycardia showed a regular narrow QRS tachycardia with a pseudo–S-wave in lead II and pseudo–R-wave in lead aVR suggestive of AVNRT.

A two-year-old child weighing 10 kg was referred for catheter ablation for drug-refractory SVT. Her mother reported a history of frequent hospitalizations despite maximal doses of oral verapamil and β-blocker medication. Electrocardiography (ECG) during sinus rhythm did not show pre-excitation. Echocardiography ruled out structural heart disease. A narrow QRS tachycardia at the rate of 260 bpm was documented during an SVT episode **(Figure 1)**. In view of recurrent hospitalization despite maximal medical management, an option of catheter ablation was discussed. Informed consent was obtained in view of the nonavailability of cryoablation and the risk of AV blockage.

**Figure 2:**
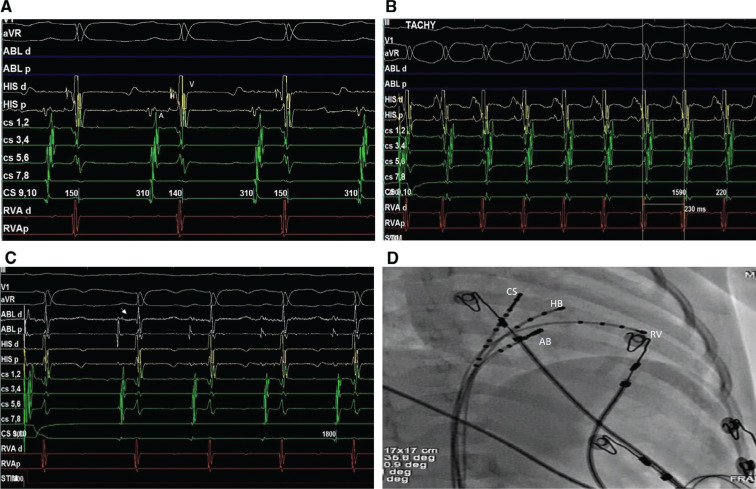
**A:** Baseline AH and HV intervals were 94 ms and 39 ms. **B**: AVNRT with a cycle length of 230 ms with a concentric activation pattern in the coronary sinus. **C:** Ablation catheter showing a fractionated atrial signal with slow-pathway potential (white arrowhead). **D:** Ablation catheter at the anterior margin of the coronary sinus in the right anterior oblique fluoroscopy view. CS: coronary sinus; HB: His bundle; AB: ablation catheter; RV: right ventricle.

RF ablation was performed under intravenous sedation. Twelve-lead ECG and intracardiac electrograms were continuously recorded using an electrophysiology system (Abbott Laboratories, Chicago, IL, USA). Venous access was obtained from both the right femoral vein and the left femoral vein. Baseline sinus rhythm showed normal AH (94 ms) and HV (39 ms) intervals **(Figure 2A)**. Tachycardia could be easily induced by programmed atrial stimulation at 400 ms (S1), 350 ms (S2), and 230 ms (S3) with an AH jump. The tachycardia cycle length was 230 ms and coronary sinus electrograms showed a concentric atrial activation pattern **(Figure 2B)**. The ventricular entrainment protocol confirmed AVNRT.

**Figure 3:**
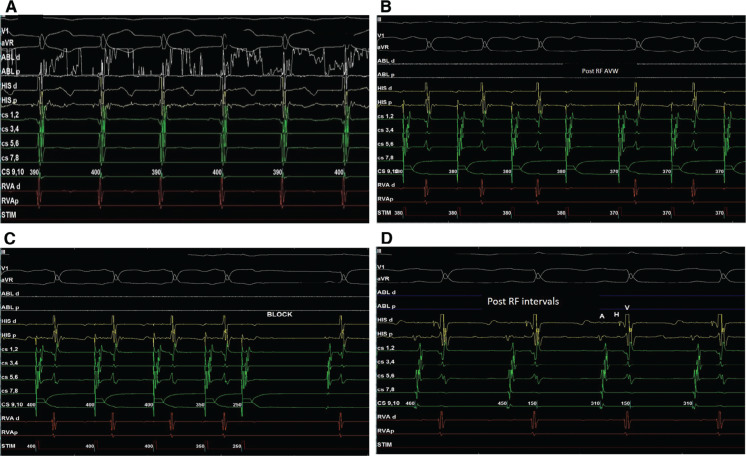
**A**: Junctional rhythm during RF ablation at a 400-ms cycle length. **B:** Post-RF ablation incremental atrial pacing showed Wenckebaching at a 380-ms cycle length. **C:** Programmed atrial stimulation showed AH block at 400 ms (S1), 350 ms (S2), and 250 ms (S3). **D:** Postablation AV-nodal conduction remained intact with AH and HV intervals of 94 ms and 40 ms.

Ablation at the posterior part of Koch’s triangle with a fractionated atrial signal with slow-pathway potential **(Figures 2C** and **2D)** using a 5-French (Fr) medium-curve RF ablation catheter (Abbott Laboratories, Chicago, IL, USA) resulted in good junctional rhythm at the 400-ms cycle length (30 W power gradually increased to 50 W, temperature of 50°C for 60 seconds) **(Figure 3A)**. The total fluoroscopy time was five minutes and the procedure lasted for 30 minutes. Postablation tachycardia could not be induced despite an aggressive induction protocol. Incremental atrial pacing showed AV Wenckebaching at a 380-ms cycle length, confirming the elimination of slow-pathway conduction **(Figure 3B)**. Fast-pathway conduction remained intact as programmed atrial stimulation revealed AH block at 400 ms (S1), 350 ms (S2), and 250 ms (S3) **(Figure 3C)**. The AH and HV intervals after ablation were 94 ms and 40 ms, respectively **(Figure 3D)**. The child was discharged the next day without any complications and remained symptom-free at 12 months after the procedure.

## Discussion

Drug-refractory SVTs are rare during childhood. The most common arrhythmias are accessory pathway–mediated AVNRT. Although there are several reports of successful ablation of orthodromic AV-reciprocating tachycardia in infants, AVNRT is rare. Our case is unique in that the AVNRT was drug-refractory despite optimal therapy and RF ablation was performed instead of cryoablation. Two major challenges exist with this protocol: (1) obtaining venous access and (2) avoiding causing injury to the AV node while ablating the slow pathway. In this present case, we restricted our venous access to the femoral vein (two punctures on each side, using a 4-Fr quadripolar catheter and a 5-Fr ablation catheter). Venous Doppler imaging was performed before discharge and ruled out venous thrombosis. To avoid AV nodal block during ablation, it was deemed safe to target fractionated atrial signals with an AV ratio of 1:10 **(Figure 2C)** and to stop ablating when there existed a fast junctional rhythm at a cycle length of less than 300 ms.

AVNRT is the second most-encountered SVT in the pediatric population. The AV node consists of two pathways: the fast pathway, which is composed of transitional cells in the region extending from the compact node to the anterior aspect of Koch’s triangle, and a slow pathway composed of a deeper inferoposterior extension.^5^ AVNRT can be safely cured by slow-pathway modification in the posterior aspect of Koch’s triangle. Cryoablation is the modality of choice in infants and children as it can reduce the incidence of AV block. However, we had to employ RF energy as cryoablation is not yet available in our region.

The most common challenge that we come across in the pediatric population is the small size of the heart and the varied anatomy associated with congenital heart diseases.^6^ The varied intracardiac and venous anatomy often makes it difficult to ablate in this population. In many centers, the procedures are modified with the usage of fewer catheters than in the adult population and the adoption of transesophageal catheters to pace the left atrium and 4-Fr ablation catheters to avoid vascular complications. The reported complications during pediatric RF ablation include complete heart block and thrombus formation. Cardiac perforation and death are rare complications and are more frequent in correlation with smaller patient sizes and left-sided procedures. Blaufox et al.^7^ showed the feasibility of catheter ablation for cardiac arrhythmias in small children weighing less than 15 kg. Of 268 RF catheter ablation procedures, 18 were performed in 14 patients weighing less than 15 kg. Orthodromic AVRT was noted in nine patients, atrial tachycardia was seen in one patient, and ventricular tachycardia was found in four patients. The complications reported included pericardial effusion, mitral regurgitation, and myocardial infarction. In the AVRT subgroup, the indexed total application time trended higher during complicated as compared with during uncomplicated procedures (40.6 versus 6.6 s/kg). The authors concluded that RF ablation can be successful in small children; however, complications appear to be related to the RF dose indexed for body size. Elsewhere, the MAP-IT registry^8^ highlighted the safety of pediatric ablation with a significant reduction in radiation exposure among the 1,417 procedures done. Cryoablation was performed in one-quarter of the cases. Two percent of the patients were younger than three years of age and their success rate and complications were not different from those of older patients.

Despite all these challenges and complications, however, catheter ablation for AVNRT is a safe procedure in the pediatric population when performed by experienced hands.

## Conclusion

AVNRT is the second most common variety of SVT in children. Drug therapy remains the first option for treating pediatric arrhythmias. In rare instances where drugs are not effective in controlling the arrhythmia, RF catheter ablation can be safely performed while safeguarding the AV node. Although single case results cannot be extrapolated to the general population, further studies could provide additional insights and confirm the safety of RF ablation in toddlers.
